# RELEASE: Preliminary results from a novel data-linkage study into mental health and substance use service utilisation by people released from Scottish prisons

**DOI:** 10.23889/ijpds.v9i5.2739

**Published:** 2024-09-18

**Authors:** Jan Savinc, Richard Kjellgren, Catriona Connell, Kate Hunt

**Affiliations:** 1 Edinburgh Napier University; 2 Scottish Centre for Administrative Data Research; 3 University of Stirling; 4 Salvation Army Centre for Addiction Services and Research; 5 Institute for Social Marketing and Health

## Objectives

People released from prison are at increased risk of dying by suicide, drug overdose, and illnesses related to drugs and alcohol. International literature demonstrates patterns in health service access that imply service accessibility may be insufficient to meet the mental health and substance use needs of this population. This study aims to compare the use of health services for mental health and substance use between people released from Scottish prisons and the general population, using national Scottish data for the first time.

## Approach

Retrospective observational cohort study, comparing all individuals released from Scottish Prison in 2015 (n=8,917), and a random general population sample (n=42,455), matched on age, sex, postcode and Scottish Index of Multiple Deprivation (SIMD) deciles. Scottish Prison Service data were linked with community prescriptions, death registrations, inpatient admissions to general and psychiatric hospitals, outpatient contacts, and emergency care.

## Results

Descriptive summaries of the demographic characteristics of the cohort and bivariate comparisons of service use across different services will be presented, alongside planned approaches using Poisson regression models of service contacts.

## Conclusions

This study advances our understanding of the feasibility of conducting research using national linked data from health and justice services and provides insight into differences in access to mental health and substance use support.

## Implications

This study advances our understanding of the feasibility of conducting research using national linked data from health and justice services and provides insight into differences in access to mental health and substance use support.

